# Selenium supplementation for patients with Graves’ hyperthyroidism (the GRASS trial): study protocol for a randomized controlled trial

**DOI:** 10.1186/1745-6215-14-119

**Published:** 2013-04-30

**Authors:** Torquil Watt, Per Cramon, Jakob Bue Bjorner, Steen Joop Bonnema, Ulla Feldt-Rasmussen, Christian Gluud, Jeppe Gram, Jane Lindschou Hansen, Laszlo Hegedüs, Nils Knudsen, Pernille Bach-Mortensen, Runa Nolsøe, Birte Nygaard, Flemming Pociot, Maria Skoog, Per Winkel, Åse Krogh Rasmussen

**Affiliations:** 1 Department of Medical Endocrinology, Copenhagen University Hospital Rigshospitalet, Blegdamsvej 9, Copenhagen, DK-2100, Denmark; 2 National Research Centre for the Working Environment, Copenhagen, Denmark; 3 Institute of Public Health Science, University of Copenhagen, Copenhagen, Denmark; 4 Department of Endocrinology and Metabolism, Odense University Hospital, Odense, Denmark; 5 Copenhagen Trial Unit, Copenhagen University Hospital Rigshospitalet, Copenhagen, Denmark; 6 Department of Endocrinology, Hospital of Southwest Denmark Esbjerg, Esbjerg, Denmark; 7 Department of Endocrinology and Gastroenterology, Copenhagen University Hospital Bispebjerg, Copenhagen, Denmark; 8 Department of Endocrinology, Copenhagen University Hospital Hvidovre, Copenhagen, Denmark; 9 Department of Cardiology and Endocrinology, Endocrine Unit, Copenhagen University Hospital Hillerød, Hillerød, Denmark; 10 Department of Internal Medicine, Endocrine Unit, Copenhagen University Hospital Herlev, Copenhagen, Denmark; 11 Glostrup Research Institute, Copenhagen University Hospital Glostrup, Copenhagen, Denmark

**Keywords:** Graves' disease, Selenium supplementation, Pragmatic trial, Quality of life, ThyPRO

## Abstract

**Background:**

Graves’ hyperthyroidism is an autoimmune disease causing hyperfunction of the thyroid gland. The concentration of selenium is high in the thyroid gland and two important groups of enzymes within the thyroid are selenoproteins, that is, they depend on selenium. Selenium may have beneficial effects on autoimmune hypothyroidism and on Graves' orbitopathy, but the effects of selenium on Graves' hyperthyroidism is unknown.

We hypothesize that adjuvant selenium may be beneficial in the treatment of Graves' hyperthyroidism. The objective is to investigate if selenium supplementation plus standard treatment with anti-thyroid drugs versus standard treatment with anti-thyroid drugs will lead to a decrease in anti-thyroid drug treatment failure (that is, failure to remain euthyroid, without further treatment, one year after cessation of anti-thyroid drug treatment), faster and longer lasting remission (that is, anti-thyroid drug treatment success), and improved quality of life in patients with Graves’ hyperthyroidism.

**Methods and design:**

The trial is an investigator-initiated, randomised, blinded, multicentre clinical trial. Inclusion criteria are: age 18 years or older; diagnosis of active Graves' hyperthyroidism within the last two months; and informed consent. Exclusion criteria are major co-morbidity; previous radioactive iodine treatment; ongoing anti-thyroid drug treatment for more than two months; treatment with immunomodulatory drugs; known allergy towards the components in the selenium and placebo pills; pregnancy or breast-feeding; and intake of selenium supplementation above 70 μg per day. We plan to include 492 participants, randomised (1:1) to two tablets of 100 μg selenium once daily for the 24 to 30 months intervention period versus two identical placebo tablets once daily.

The primary outcome is the proportion of participants with anti-thyroid drug treatment failure (see above) at the end of the intervention period (24 to 30 months). Secondary outcomes are: thyroid-specific quality of life during the first year after randomisation; level of thyroid stimulating hormone-receptor antibodies at 18 months after randomisation and at the end of the intervention period (24 to 30 months); hyperthyroid symptoms during the first year after randomisation; eye symptoms during the first year after randomisation, and at the end of the intervention period (24 to 30 months); adverse reactions during the intervention period; and serious adverse events during the intervention period.

**Discussion:**

It was of great importance to the initiators of this trial, that the results would be directly applicable to daily clinical practice. Therefore, it was designed as a pragmatic trial: the patients follow their usual treatment at their usual hospitals. In order to still collect high quality data on the clinical course and quality of life, an elaborate trial management system was designed to keep track of patient input, need for trial personnel input and action, and to collect data from medical chart systems. Meticulous follow-up on missing responses to the QoL measurements has been incorporated into the system, to minimise missing quality of life data. Monitoring of adverse reactions and events is achieved by thorough instruction of the participants, surveillance of patient-reported outcomes, and integration with national databases regarding hospitalizations. A very long intervention period was necessary, since patients are not considered in remission until one year after stopping anti-thyroid drugs. Usually, patients are treated for 12 to 18 months with anti-thyroid drugs, yielding a total intervention period of 24 to 30 months.

**Trial registration:**

ClinicalTrials.gov: NCT01611896.

## Background

Graves’ hyperthyroidism is an autoimmune disease causing hyperfunction of the thyroid gland by a mechanism, where auto-antibodies bind to and stimulate the thyroid stimulating hormone (TSH) receptor. It affects individuals of all ages and is five to ten times more common in women than in men, with an overall annual incidence in Denmark of about 1,700 patients in 5.5 million people [1]. Treatment of Graves’ hyperthyroidism comprise: 1) anti-thyroid drugs (ATD) (for example, methimazole or propylthiouracil); 2) thyroidectomy, that is, surgical removal of the thyroid gland, and 3) radioactive iodine treatment (I^131^), which permanently reduces thyroid function. In Europe, the primary treatment is usually ATD, which leads to resolution of hyperthyroidism (that is, euthyroidism) in 85% to 90% of patients within 6 weeks [2]. This treatment is usually continued for a period of 12 to 18 months and then tapered off (ATD treatment withdrawal). However, 30% to 60% of patients will experience relapse of hyperthyroidism during the following years [2]. Patients with Graves' hyperthyroidism suffer from a wide range of symptoms and have major impairments in most areas of quality of life (QoL) [3].

Selenium is an essential trace element important for human health. The main dietary sources of selenium in Denmark are meat, poultry, dairy products, bread, cereals and fish [4,5]. The recommended daily intake of selenium is 40 μg for women and 50 μg for men. The estimated actual daily intake in Denmark is considered sufficient, but it cannot be ruled out that about 10% of the Danish population could benefit from selenium supplementation [4]. The upper tolerable level of selenium intake is set to 400 μg per day in the USA [6] and 300 μg per day in the EU [7].

The thyroid gland has the highest selenium concentration per unit weight among all tissues. Selenium is incorporated into key enzymes involved in several metabolic pathways. The main selenoprotein families are the glutathione peroxidases, the thioredoxin reductases and the iodothyronine deiodinases [5]. It is hypothesized that the glutathione peroxidases and the thioredoxin reductases participate in a complex defence system maintaining normal thyroid function by protecting the gland from both hydrogen peroxide, which is produced by the thyrocytes, and reactive oxygen intermediates [8,9]. It has thus been hypothesized, that selenium may have a beneficial role in autoimmune thyroid diseases, by blunting the autoimmune process.

We have not identified any published randomised trials of selenium supplementation in patients with Graves’ hyperthyroidism. On ClinicalTrials.gov, one ongoing randomised trial is registered on selenium supplementation in Graves’ thyrotoxicosis (NCT01247077). This trial assesses neuropsychological well-being (not otherwise specified), after nine months intake of 200 μg selenium, and includes 44 participants. In a multicentre trial among 159 patients with mild Graves' orbitopathy, without hyperthyroidism, 200 μg selenium selenite improved disease-specific QoL (*P* <0.001) and reduced eye disease severity (*P =* 0.01) [10]. Another trial evaluated the effect of adding a mixture of antioxidants, including 60 μg of selenium (not otherwise specified) to standard ATD treatment in 29 patients with Graves' disease. During the 60-day follow up period euthyroidism was reached more rapidly in patients receiving antioxidants [11-13]. In contrast, several randomised trials have evaluated the effect of selenium on the other major autoimmune thyroid disease: autoimmune hypothyroidism. In six [14-19] of seven [14-20] placebo-controlled clinical trials, selenium treatment reduced thyroid peroxidase antibody (TPOAb) levels, indicating a beneficial effect on the autoimmune activity.

We hypothesize that the addition of selenium supplementation to the standard treatment with ATD in patients with active Graves’ hyperthyroidism will lead to a decrease in ATD treatment failure (that is, fewer patients with relapse), faster remission, and improved quality of life.

## Methods and design

### Objectives

The primary objective is to investigate the effect of selenium supplementation on the proportion of participants with ATD treatment failure, that is, failure to remain euthyroid without further treatment one year after cessation of ATD treatment.

The secondary objectives are to investigate the effect on thyroid-specific QoL, level of TSH-receptor antibody (TRAb), hyperthyroid symptoms, eye symptoms, adverse reactions, and serious adverse events. Further, we wish to explore the effect of selenium on ATD treatment duration, incidence of Graves' orbitopathy, and hypothyroid symptoms.

### Design

**Figure 1:**
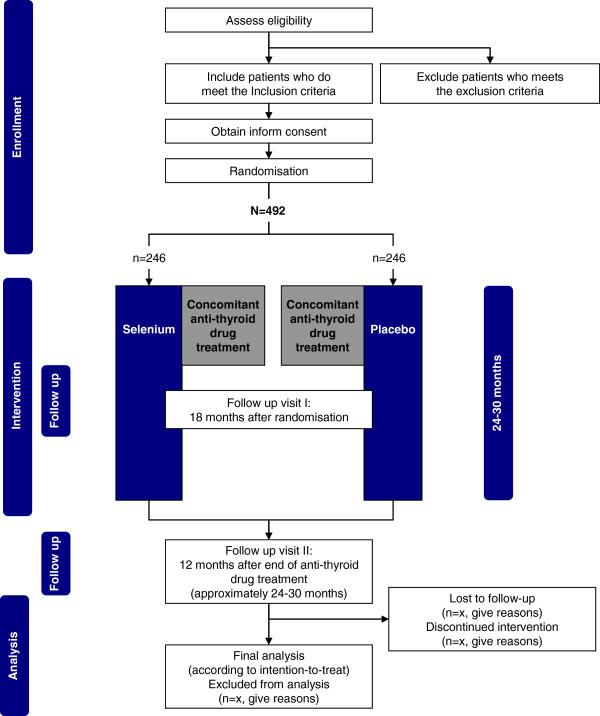
Trial flow chart.

The GRASS (GRAves’ disease Selenium Supplementation trial) trial is an investigator-initiated, randomised, blinded, multicentre clinical trial of selenium supplementation versus placebo in patients with Graves’ hyperthyroidism. The trial has a parallel-arm design with 1:1 allocation to the experimental intervention group and the control intervention group, and involving seven clinical trial sites in Denmark (see Figure 1). The trial also includes a questionnaire and register-based follow up period up to ten years after completion of the intervention period.

### Trial participants

All patients with current hyperthyroidism, who are referred to or being followed at the participating clinical trial sites, are considered for participation. Patients are eligible for the GRASS trial, if they comply with the following inclusion and exclusion criteria.

#### Inclusion criteria

The inclusion criteria are: Graves’ hyperthyroidism (first-time diagnosis, defined as patients not yet receiving ATD treatment, or having received ATD treatment continuously for less than two months, or relapse of Graves’ hyperthyroidism defined as patients previously having received and discontinued treatment with ATD); active Graves' hyperthyroidism (TSH <0.1 mU/L and positive TRAb according to local laboratory results) measured within the last two months prior to the inclusion date; age 18 years or older; provision of written informed consent.

#### Exclusion criteria

The exclusion criteria are: major co-morbidity, rendering the participants unlikely to continuously receive the trial intervention; previous treatment with radioactive iodine; ongoing ATD treatment for more than two months; treatment with immunomodulatory drugs, such as cyclosporine A, methotrexate, cyclophosphamide; allergy to the components in the selenium and placebo pills; pregnancy or breast-feeding; intake of selenium supplementation above 70 μg per day; inability to read and understand Danish; lack of provision of informed consent.

### Trial intervention

#### Selenium

The compound used in this trial is organic selenium, in the form of selenium yeast, which mainly consists of selenomethionine. The specific product is Organisk selen, 100 μg tablets and is produced by Jemo-Pharm A/S, Stege, Denmark (http://www.jemo-pharm.dk/frame.cfm/cms/id=977/sprog=2/grp=6/menu=1/). The daily dose is set at 200 μg (two tablets taken in the morning). The trial dosage is based on the available clinical data, is not considered to cause adverse reactions, and is lower than the upper tolerable intake level of 300 μg per day [6,7].

#### Placebo

Placebo tablets, identical in size, appearance, taste, smell, and solubility to the experimental intervention tablet are produced by Jemo-Pharm A/S. They have the same content as Organisk selen but are without selenium, as the selenium yeast has been exchanged with yeast grown in selenium-deplete media. The placebo regimen is identical to the selenium regimen.

#### Randomisation

Randomisation will be performed centrally. The allocation sequence is computer-generated with a varying block size kept unknown to the investigators. Randomisation is stratified by clinical trial site and disease status (incident or relapse), and the allocation ratio is 1:1.

#### Informed consent procedure

Potential participants are identified at referral or visits to the outpatient clinics and include all patients with current hyperthyroidism (that is, elevated thyroid hormone levels). Where Graves’ hyperthyroidism is confirmed (that is, TRAb is positive), the potential participant is invited to an information visit by letter. The visit consists of obtaining history, blood sampling, information about trial contact, and randomisation.

#### Duration

**Figure 2:**
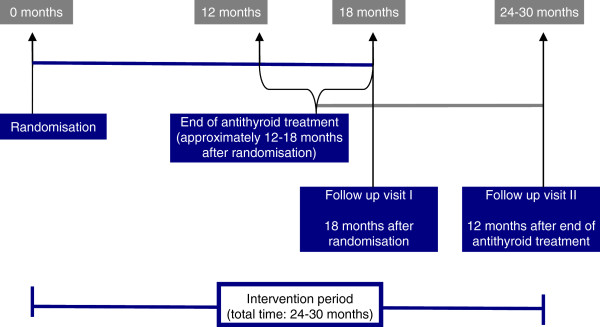
Duration of the intervention.

The intervention period for each participant will be 24 to 30 months, as selenium will be given until 12 months after cessation of ATD treatment, which usually lasts 12 to 18 months (Figure 2). The total trial duration is expected to be about 4 years (inclusion period about 17 months and up to 30 months intervention period).

#### Concomitant medication or treatment

The trial participants receive conventional ATD treatment. Treatment of hyperthyroidism, will take place within the participating clinical trial sites, according to the clinical standards set by the departments. ATD treatment withdrawal must be considered or attempted 18 months after randomisation at the latest.

Participants are advised not to take extra selenium supplementation during the trial. A dose of <70 μg per day is allowed (the content in multivitamin tablets is 55 μg). The participants’ consumption of additional selenium supplements will be monitored during the trial through self-report forms.

#### Monitoring for intervention compliance

Participant compliance with the intervention will be monitored by self-reported tablet intake at 6 and 12 weeks, and 6, 12 and 24 months (Table 1), and by tablet-counting at trial visits at 18 months and at the end of the intervention (24 to 30 months). An investigator will contact the patient if the trial data management system flags a participant as non-compliant (or overdosing), according to self-report of the number of unopened containers.

**Table 1:** Trial schedule for assessments

		**Follow up**						
**Variable**	**Inclusion (baseline)**	**6 weeks**	**12 weeks**	**6 months**	**12 months**	**18 months**	**24 months**	**12 (± 1) months after ATD treatment withdrawal**^**†**^
Visit	x					x		x
ATD treatment						x	x	x
Thyroid function	x^1^					x^1^		x^1^
TSH-Receptor Antibodies	x^1^					x_s_		x_s_
Serum selenium	x					(x_s_)		(x_s_)
Creatinine/iodine ratio in spot urine	x							
Storage samples (blood and urine)	x					x		x
ThyPRO	x_r_	x_r_	x_r_	x_r_	x_r_	x_r_	x_r_	x_r_
Tablet count		x_r_	x_r_	x_r_	x_r_	x_r_	x_r_	x_r_
Consumption of additional selenium				x_r_	x_r_	x_r_	x_r_	
Adverse events*		x_r_	x_r_	x_r_	x_r_	x_r_	x_r_	x_r_
Serious adverse events								x
Referral to ablative therapy						x		x

All assessments must be made at the time points specified above. If the assessment is not possible at the specified time, the assessment shall still be conducted, and the time shall be noted in the electronic case report form (eCRF). Following this, deviations from the protocol can be assessed. ^1^As part of usual clinical practice (f = free (non-protein bound)), that is, all measured analyses are collected continuously from medical systems; x_s_, analysed on stored plasma/serum samples after trial completion; x_r_, self-reported data by participant; ^†^participants for whom ATD treatment withdrawal has not been attempted, or has been unsuccessful, will be followed up at 36 months after randomisation (± 1 month). ATD, anti-thyroid drugs; TSH, thyroid stimulating hormone; ThyPRO, thyroid patient-reported outcome.

#### Discontinuation

A participant who no longer wishes to participate in the trial can withdraw his/her informed consent at any time without need of further explanation, and this will not have any consequences for the participant’s further treatment. In order to conduct intention-to-treat analyses with as few missing data as possible, the investigator may ask the participants which aspects of the trial, they wish to withdraw from. These can include the following: receipt of the trial intervention; participation in the remaining follow up assessments, and analysis of data already collected. The investigators will discontinue a participant’s taking of the trial intervention at any time, if the participant: experiences intolerable adverse reactions; is diagnosed with any of the exclusion criteria during the intervention period; is referred for ablative therapy (radioactive iodine or thyroid surgery) during the intervention period. In all three cases, the investigator and/or the treating physician will, if possible, encourage the participant to continue with follow up assessment and to allow the use of collected data in the analyses.

#### Blinding

Blinding will be maintained for all parties in the trial, throughout all aspects of the trial. The trial interventions will be identical, and will be packed in identical packages by the Capital Region Pharmacy, and therefore, knowledge of allocated intervention group will be unknown to participants and investigators. All outcome assessments will be performed blinded and statistical analyses will be performed with the blinding intact.

#### Safety

Selenium is tolerated in short-term doses up to 10,000 μg (that is, 100 experimental GRASS tablets). Acute intoxication is very rare and has only been related to accidental or suicidal intake [4,21,22]. Chronic intoxication requires long-term intake of at least 800 μg per day [23], according to epidemiological studies in areas with very high selenium content in the soil. Thus, no signs of chronic intoxication have been observed in areas with daily intakes of up to about 800 μg daily. In geographically highly exposed groups without signs of intoxication the serum concentrations have been reported to be 148 to 363 μg per L [24] and 284 to 472 μg per L [23], respectively. In a study of 200 μg selenium supplementation per day, the serum concentration reached a plateau at around 190 μg per L [25].

According to the Danish National Food Institute, the 99th percentile for daily selenium intake through diet in Denmark is 93 μg for men and 72 μg for women. Therefore, a dosage of 200 μg per day should not bring trial participants above the established upper tolerable intake of 300 μg per day. A review of the safety of selenium supplementation with selenium yeast, which will be used in the GRASS trial [26], concludes that in about a dozen supplementation studies, none has shown evidence of toxicity even up to an intake of 800 μg selenium per day over a period of years. In conclusion, the experimental intervention with 200 μg selenium per day is not expected to cause adverse reactions. Regardless, participants will be monitored for adverse events.

Overdosing can lead to gastrointestinal discomfort, hair and nail malformations and loss, peripheral neurological symptoms, fatigue and dizziness, and, in the case of very large selenium loads (1,000 times the daily trial dose), cardiovascular collapse and respiratory distress [21,22].

Assessment and reporting of adverse reactions (ARs): participants are prompted to self-report ARs at 6 and 12 weeks, and 6, 12 and 24 months, and are questioned about ARs at the study visits at 18 months of treatment and 12 months after stopping ATD treatment, respectively. ARs will be reported as a trial outcome. In addition, participants are instructed to contact their trial contact person if they experience symptoms suggestive of ARs.

Assessment and reporting of serious ARs (SARs), serious suspected SARs (SUSARs) and serious adverse events (SAEs): data on hospital admissions and mortality will be obtained through national registries at the end of the trial. Also, participants are informed and instructed to contact their trial contact person if they are admitted to a hospital for selenium intoxication, experience a clinical picture indicative of selenium intoxication, or experience a clinical picture that is unexpected but suspected to be related to selenium intoxication. When a possible serious event (SAE, SAR, or SUSAR) is identified, details will be sought from the patient’s medical record and through direct contact with the patient. Any SAE, SAR or SUSAR will be reported as an outcome measure.

### Outcomes

Outcomes will be assessed seven times during the trial (Table 1).

#### Primary outcome

The primary outcome is the proportion of participants with the composite outcome of ATD treatment failure in participants receiving ATD treatment during the last 12 months (± 1 month) of the intervention period, who have had thyroid hyperfunction (TSH <0.1) during the last 12 months (± 1 month) of the intervention period, or have been referred for ablative therapy (radioactive iodine or thyroid surgery) at some point during the entire intervention period.

#### Secondary outcomes

The secondary outcomes are each component of the primary outcome as follows: proportion of participants who receive ATD treatment (at any level) during the last 12 months (± 1 month) of the intervention period (separate component of the primary outcome); proportion of participants who have thyroid hyperfunction (TSH <0.1) during the last 12 months (± 1 month) of the intervention period (separate component of the primary outcome); proportion of participants who have been referred to ablative therapy (radioactive iodine or thyroid surgery) at some point during the entire intervention period (separate component of the primary outcome); thyroid-specific QoL during the first year after randomisation, and at the end of the intervention period (24 to 30 months), as measured by the global score in the ThyPRO questionnaire (Appendix 1); level of TRAb at 18 months, and at the end of the intervention period (24 to 30 months); hyperthyroid symptoms (ThyPRO subscale) during the first year after randomisation; eye symptoms (ThyPRO subscale) during the first year after randomisation, and at the end of the intervention period (24 to 30 months); number of patients with ARs during the intervention period, and number of patients with serious adverse events during the intervention period.

#### Exploratory outcomes

The following outcomes are of an exploratory nature: time to ATD treatment withdrawal (unsuccessful participants will be censored at 18 months); cost-effectiveness of the experimental intervention; incidence of Graves' orbitopathy during the intervention period, assessed as clinical activity score (CAS) >1 among patients with CAS scoring in the medical chart, and hypothyroid symptoms (ThyPRO subscale) during the intervention period.

### The trial data management system

As a result of the pragmatic design with minimal participant-trial interaction, a large part of the data collection, trial conduct, and trial surveillance and timing is handled by the trial data management system. This trial data management system consists of a patient-survey-interface, a trial-personnel-interface, a system-integration interface and a programme *motor*. The system will be used for collection of outcomes, adverse events, and other trial-relevant information; for timing of trial events, that is, time for patient-reported outcomes and trial visits; for identification of need for actions (for example, contact to a participant); and for delivery of output to personnel or participants (for example, email notifications).

### Research biobank

A research biobank for serum/plasma and urine samples will be established. The samples will initially be kept at each clinical trial site, and will later be analysed at central laboratories. Participants are informed verbally and in writing, and will consent to the withdrawal and storing of biological material in the GRASS trial. Any remaining samples will be transferred to a Biobank designated for future use. These samples will be used for genome-wide association studies (of predictors for remission and experimental intervention effects, or other indicators of autoimmunity), as may be specified in forthcoming protocols.

### Monitoring

The trial will be monitored according to the International Committee of Harmonization (ICH) guidelines for good clinical research practice [27] by internal monitoring.

### Statistical analysis

#### Primary outcome: ATD treatment failure - sample size estimation

Prior data indicate that the proportion of patients in the placebo group with ATD treatment failure is 50% [28]. If the true proportion with ATD treatment failure is 37.5% among selenium-treated participants (that is, a relative risk reduction of 25%), we will need to include 492 participants (246 experimental and 246 control participants) to be able to reject the null hypothesis with a power of 80% and a risk of type I error of 5%.

The patient catchment areas of the participating centres include 1,640,000 persons. Assuming an annual incidence of Graves' hyperthyroidism of 40 per 100,000 after iodine fortification [1,29] and 50% recurrence rate, the incident population (including recurrences) will be 788 patients annually. Assuming an inclusion of 45% of the potential patients, this will lead to about 29 participants per month and an inclusion period of about 17 months.

#### Secondary outcomes - power estimation

For the ThyPRO Global score, hyperthyroid symptoms and eye symptoms, if the true difference between experimental and the control participants is 5 (on a scale of 0 to 100) with SD of 20, we will be able to reject the null hypothesis that the population means of the experimental and control groups are equal with a probability (power) of 79%. The associated type I-risk is 5%.

For the level of TRAb, if the true difference in TRAb between experimental and control participants is 0.15 IU per L with SD of 0.5 IU per L, we will be able to reject the null hypothesis with a probability (power) of 91% and a type I error-risk of 5%.

For the number of patients with adverse reactions, prior data indicate that the proportion of participants who experience adverse reactions in the control group is about 5% [26]. If the true proportion of participants in the experimental group who experience adverse reactions is 10%, we will be able to reject the null hypothesis that the failure rates for experimental and control participants are equal with probability (power) 50%. The type I error probability associated with this test is 5%.

#### Data analysis

All analyses will be intention-to-treat analyses with the intervention group concealed until two conclusions are drawn. The significance test will be at the 5% level and two-sided. Table 2 shows the priority for each outcome, when it will be measured, the mathematical type of measure, and the analytical procedure to be used when analyzing the outcome values.

**Table 2:** Outcome measures, their priorities, times of measurement, mathematical types and analytical categories (defining the statistical analysis to which they will be subjected)

**Outcome**	**Times of measurements**	**Type of quantity**	**Regression analysis to be applied**
Primary outcome			
ATD treatment failure	End of trial	Binary	Logistic regression
Secondary outcomes			
1. ATD treatment within last 12 months	End of trial	Binary	Logistic regression
2. Thyroid hyperfunction after ATD treatment withdrawal	End of trial	Binary	Logistic regression
3. Ablative therapy	End of trial	Binary	Logistic regression
4. Global QoL ThyPRO score	a) Time sequence of five measurements^†^	Numerical	a) Mixed-model with repeated measures (MMRM)
	b)12 months following ATD treatment withdrawal^‡^		b) General linear univariate model. As sensitivity analysis: Mann-Whitney test
5. Level of TRAb	After 18 months and at the end of intervention period	Numerical	General linear univariate model. As sensitivity analysis: Mann-Whitney test
6. ThyPRO - hyperthyroid symptoms	a) Time sequence of five measurements^†^	Numerical	a) Mixed-model with repeated measures (MMRM)
	b) 12 months following ATD treatment withdrawal^‡^		b) General linear univariate model. As sensitivity analysis: Mann-Whitney test
7. ThyPRO - eye symptoms	a) Time sequence of five measurements^†^	Numerical	a) Mixed-model with repeated measures (MMRM)
	b) 12 months following ATD treatment withdrawal^‡^		b) General linear univariate model. As sensitivity analysis: Mann-Whitney test
8. Adverse reactions	End of trial	Rate = count/period of intervention/day	Generalised linear model, Poisson distribution, link = log. As sensitivity analysis: Mann-Whitney test
9. Serious adverse reactions	End of trial	Rate = count/period of intervention/day	Generalised linear model, Poisson distribution, link = log. As sensitivity analysis: Mann-Whitney test
Exploratory outcomes			
1. Time to ATD withdrawal ^§^	End of trial	Numerical (time until ATD withdrawal (or censoring))	Cox proportional hazard rate model.As sensitivity analysis: Kaplan-Meier estimates of survival function
2. Incidence of Graves’ orbitopathy - CAS score	End of trial	Binary (CAS >1)	Logistic regression
3. ThyPRO - Hypothyroid symptoms	a) Time sequence of five measurements^†^	Numerical	a) Mixed-model with repeated measures (MMRM)
	b) 12 months following ATD treatment withdrawal^‡^		b) General linear univariate model. As sensitivity analysis: MannWhitney test

^†^This analysis (2) includes four measurements relative to the reference time (6 weeks, 12 weeks, 6 months and 12 months). ^‡^This analysis (3) includes one measurement (12 months following ATD treatment withdrawal or failure to withdraw (alternative treatment instituted or deadline expired)) relative to a different reference time. ^§^End of ATD treatment or censoring at 18 months after randomisation or ablative surgery, provided the latter takes place during ATD treatment and prior to time of censoring. ATD, anti-thyroid drugs; QoL, quality of life; ThyPRO, thyroid patient-reported outcome; TRAb, TSH-receptor antibodies; CAS, clinical activity score.

#### Analytical procedures

Depending on the specific type of outcome measure, one of five types of regression analysis will be applied (Table 2). Indicator variable I (1 if X and 0 if Y) is included as a covariate and the outcome measure (y) as the dependent variable. All analyses will be conducted both as unadjusted analyses and adjusted for stratification variables (clinical trial site and disease status (incident or relapse)). Discrepancies between the results of the two analyses will be discussed. For each covariate, an exploratory analysis, including the interaction between the covariate and the intervention indicator, will be conducted.

If the distribution of the primary outcome measure differs significantly between the two intervention groups, and the percentage of missing values is larger than 5%, multiple imputations (MI) will be used. If so, the result obtained by imputation will be the primary result. In all events, the potential for bias caused by non-random missing values will be assessed using a worst- and best-case scenario. The gate keeping method of Dmitrienko *et al*. [30] will be used to adjust the observed *P*-values.

### Ethical considerations

The GRASS trial has been approved by the Regional Ethics Committee (H-4-2012-026). The trial will be conducted in compliance with the guidelines of the latest Declaration of Helsinki and the International Conference on Harmonization (ICH) Good Clinical Practice (GCP) Guidelines [27].

### Publication plan

The aim is to publish all results, positive, neutral, and negative, in peer-reviewed international journals. Authorship will be determined according to the guidelines from the International Committee of Medical Journal Editors [31].

## Discussion

The design of the GRASS trial has faced three major challenges: the intent to conduct it as a pragmatic trial with as little interference with daily clinical management as possible, while still measuring QoL meticulously; the intent to comply with the ICH guidelines for GCP, even though the intervention is widely considered to be non-toxic and rather, a supplemental nutrient than a drug; and the need for a very long intervention period.

It was of great importance to the initiators of this trial, that the results would be directly applicable to daily clinical practice. Therefore, it was decided to conduct a pragmatic trial [32], with as little interference with daily clinical practice as possible. This could be accomplished by letting the patients follow their usual treatment at their usual hospitals by whichever physician was involved in their treatment. At the same time, we found it important to collect high quality data on the clinical course and QoL. We have, therefore, put great effort into the design of a trial management system, which could solve this schism of distance from participants compared to close monitoring. The system initiates and keeps track of patient input (QoL-measurements and other patient-reported outcomes), identifies need for trial personnel input and action, and collects data on thyroid function from medical chart systems. The system also identifies the site of the information-provider, as well as the disease status of new participants, and delivers randomisation codes stratified by site and disease status. Meticulous follow up on missing responses to the QoL measurements is incorporated into the system, to minimise the usual major problem with missing QoL data in clinical trials.

Somewhat similarly, the need to monitor adverse reactions and events in accordance with the ICH guidelines was in conflict with the intent to interfere as little as possible and with the fact that no previous selenium trial has identified any adverse reactions, which would also be quite surprising, given the wide therapeutic range of this dietary supplement. This is solved by a combination of thorough instruction of the participants to contact their trial person in case of symptoms indicative of adverse reactions or events, surveillance of patient-responses to prompts through the trial management system, and integration with national databases regarding hospitalizations.

The third issue was the decision to continue trial intervention until one year after stopping anti-thyroid drugs (ATD treatment withdrawal). Patients are not considered in remission unless they are still euthyroid one year after stopping medication. Since selenium is considered to have an attenuating effect on thyroid autoimmunity, a long duration of treatment was considered necessary to test our hypothesis, that is, that selenium can indeed lead to more patients staying in remission (and not just reaching euthyroidism faster and obtaining better QoL).

## Trial status

The first patient was enrolled in December 2012.

## piations

ATD: Anti-thyroid drug; AR: Adverse reaction; CAS: Clinical activity score; CRF: Case report form; GCP: Good Clinical Practice; ICH: International Conference on Harmonization; QoL: Quality of life; SAE: Serious adverse event; SAR: Serious adverse reaction; s-Se: Serum selenium; SmPC: Summary of products characteristics; SUSAR: Suspected serious adverse reaction; T3: Triiodothyronine; T4: Thyroxine; ThyPRO: Thyroid patient related outcome (thyroid-specific quality of life questionnaire); TPOAb: Thyroid peroxidase antibody; TSH: Thyroid stimulating hormone; TRAb: Thyroid stimulating hormone receptor antibody (TSH receptor antibody).

## Competing interest

None of the investigators have any financial or non-financial competing interest.

## Author contributions

All authors contributed to the design of the trial, preparation and review of the manuscript and all authors read and approved the final manuscript.
